# Supplementary material for: Deep learning-based automatic image classification of oral cancer cells acquiring chemoresistance in vitro
Source: PLoS One. 2024 Nov 1;19(11):e0310304. doi: 10.1371/journal.pone.0310304 (PMC11530068; doi:10.1371/journal.pone.0310304)
Supplement: Attachment — Submitted filename: Comments to authors.pdf [file pone.0310304.s001.pdf]

Comments to authors

The research investigates the use of CNN to distinguish between different cell types and hopes to identify chemoresistant cancer cells based on their morphological features.

The manuscript has potential but needs further work to be published.

Please revise, clarify, or address the following:

-The current title may be construed as misleading since it implies that the image analysis is completed on oral cancer tissue and inflates the clinical significance of the paper. Usage of cell lines for image analysis is also not mentioned at all in the abstract.

- HEK293T cells ("CONTROL") – why choose kidney cells for control instead of oral keratinocytes?

-To my knowledge, there is no gold-standard in assessing for chemoresistence. Please explain how you overcame this challenge to apply your ground truth labels.

-Would be helpful if a table with all the CNN training parameters were included (learning rate, epoch, batch size, ect)

- The authors reported that the test set showed a perfect score of 100% accuracy. This is unusual unless the data is incredibly homogenous which cell lines may be. Can you add prediction results when the model is applied to different oral cancer cell lines that were not originally trained on? This would be a much better surrogate for the heterogeneity seen in actual patient tissue samples and prove the model's generalizability- even if accuracy is lower.

-Conclusion section: line 409 "which is a key feature of cancer cells in response to tumor microenvironment by dynamic cell state transition." This phrase is confusing. The biologic processes behind tumor heterogeneity is complex and has to do with more than the tumor microenvironment.

-Line 410- What does "dynamic cell state transition" mean?

-Gradcam is used to visualize the predictions but what morphology is expected in chemoresistent cells? Is this matching the Gradcam areas?

-
