# Supplementary material for: Deep learning-based automatic image classification of oral cancer cells acquiring chemoresistance in vitro
Source: PLoS One. 2024 Nov 1;19(11):e0310304. doi: 10.1371/journal.pone.0310304 (PMC11530068; doi:10.1371/journal.pone.0310304)
Supplement: Attachment — Submitted filename: Response to Reviewers.docx [file pone.0310304.s002.docx]

July 23, 2024

Dear Editor,

We are very grateful to have the opportunity to revise our manuscript entitled “Automatic image classification for the morphological shape of oral cancer cells in vitro by deep learning” (revised to “**Deep learning-based automatic image classification of oral cancer cells acquiring chemoresistance in vitro**”).

We greatly appreciate the reviewers’ comments and have revised the manuscript accordingly. Our responses are given in a point-by-point manner below.

We hope our responses are satisfactory and the revised manuscript is appropriate for publication.

Thank you very much for your kind consideration of our manuscript.

**Comments from Editor:**

Comment #1:

When submitting your revision, we need you to address these additional requirements.

Please ensure that your manuscript meets PLOS ONE's style requirements, including those for file naming. The PLOS ONE style templates can be found at

https://journals.plos.org/plosone/s/file?id=wjVg/PLOSOne_formatting_sample_main_body.pdf and

https://journals.plos.org/plosone/s/file?id=ba62/PLOSOne_formatting_sample_title_authors_affiliations.pdf.

Response #1:

We thank the Editor’s reminder and have ensured that our manuscript meets PLOS ONE’s requirements.

Comment #2:

Please note that PLOS ONE has specific guidelines on code sharing for submissions in which author-generated code underpins the findings in the manuscript. In these cases, all author-generated code must be made available without restrictions upon publication of the work. Please review our guidelines at https://journals.plos.org/plosone/s/materials-and-software-sharing#loc-sharing-code and ensure that your code is shared in a way that follows best practice and facilitates reproducibility and reuse.

Response #2:

We thank the Editor’s suggestion and have shared our code to facilitate reproducibility of our results.

Comment #3:
We note that the grant information you provided in the ‘Funding Information’ and ‘Financial Disclosure’ sections do not match.

When you resubmit, please ensure that you provide the correct grant numbers for the awards you received for your study in the ‘Funding Information’ section.

Response #3:

We thank the Editor’s suggestion and have corrected the grant numbers in the ‘Funding Information’ in cover letter.

Comment #4:
Thank you for stating the following financial disclosure:

[National Science and Technology Council, Taiwan].

Please state what role the funders took in the study. If the funders had no role, please state: "The funders had no role in study design, data collection and analysis, decision to publish, or preparation of the manuscript."

If this statement is not correct you must amend it as needed.

Please include this amended Role of Funder statement in your cover letter; we will change the online submission form on your behalf.

Response #4:

We thank the Editor’s suggestion to clarity the role of the funder and have included the statement “The funders had no role in study design, data collection and analysis, decision to publish, or preparation of the manuscript” in cover letter.

Comment #5:
Thank you for stating the following in the Acknowledgments Section of your manuscript:

[This work was supported in part by the National Science and Technology Council,

Taiwan, R.O.C., under Grant No. MOST-112-2634-F-A49-003,

MOST-112-2321-B-075-002, MOST-112-2321-B-A49-021, and

MOST-110-2118-MA49-002-MY3 in part by the Higher Education Sprout Project of the

National Yang Ming Chiao Tung University and Ministry of Education, Taiwan, R.O.C.,

and in part by Ministry of Education Yushan Scholar Program, Taiwan, R.O.C. We are

grateful to the National Center for High-performance Computing for computer time and

facilities.]

We note that you have provided funding information that is not currently declared in your Funding Statement. However, funding information should not appear in the Acknowledgments section or other areas of your manuscript. We will only publish funding information present in the Funding Statement section of the online submission form.

Please remove any funding-related text from the manuscript and let us know how you would like to update your Funding Statement. Currently, your Funding Statement reads as follows:

[National Science and Technology Council, Taiwan].

Please include your amended statements within your cover letter; we will change the online submission form on your behalf.

Response #5:

We thank the Editor’s reminder to remove the funding information in the manuscript. We have updated the funding statement in the amended cover letter.

Comment #6:
We note that Figure(s) 1, 3, and 7 in your submission contain copyrighted images. All PLOS content is published under the Creative Commons Attribution License (CC BY 4.0), which means that the manuscript, images, and Supporting Information files will be freely available online, and any third party is permitted to access, download, copy, distribute, and use these materials in any way, even commercially, with proper attribution. For more information, see our copyright guidelines: http://journals.plos.org/plosone/s/licenses-and-copyright.

We require you to either (1) present written permission from the copyright holder to publish these figures specifically under the CC BY 4.0 license, or (2) remove the figures from your submission:

a. You may seek permission from the original copyright holder of Figure(s) 1, 3, and 7 to publish the content specifically under the CC BY 4.0 license.

We recommend that you contact the original copyright holder with the Content Permission Form (http://journals.plos.org/plosone/s/file?id=7c09/content-permission-form.pdf) and the following text:

“I request permission for the open-access journal PLOS ONE to publish XXX under the Creative Commons Attribution License (CCAL) CC BY 4.0 (http://creativecommons.org/licenses/by/4.0/). Please be aware that this license allows unrestricted use and distribution, even commercially, by third parties. Please reply and provide explicit written permission to publish XXX under a CC BY license and complete the attached form.”

Please upload the completed Content Permission Form or other proof of granted permissions as an ""Other"" file with your submission.

In the figure caption of the copyrighted figure, please include the following text: “Reprinted from [ref] under a CC BY license, with permission from [name of publisher], original copyright [original copyright year].”

b. If you are unable to obtain permission from the original copyright holder to publish these figures under the CC BY 4.0 license or if the copyright holder’s requirements are incompatible with the CC BY 4.0 license, please either i) remove the figure or ii) supply a replacement figure that complies with the CC BY 4.0 license. Please check copyright information on all replacement figures and update the figure caption with source information. If applicable, please specify in the figure caption text when a figure is similar but not identical to the original image and is therefore for illustrative purposes only.

Response #6:

We thank the Editor for inquiring about the copyrights of the Figure(s) 1, 3, and 7. In fact, these figures are generated by our own team, and therefore, they are not subject to any copyright restrictions. They can be freely published under the Creative Commons Attribution License (CC BY 4.0), allowing unrestricted use, distribution, and reproduction with proper attribution.

**Reviewer #1**

Comment #1-1:
The authors applied a neural network to predict differences in morphological characteristics between two different cell lines (oral squamous cell carcinoma cells and a control kidney cell line - why not oral keratinocytes as control?). Though their methodological design was thoughtful and creative, it has limited clinical significance since the experiments were performed in two cell lines and does not capture the histomorphology spectrum of oral cancer cells.

Response #1-1:
We thank the reviewer’s comment. The primary aim of this study is to investigate whether CNN can effectively perceive changes in cell morphology, particularly when cells acquire features such as chemoresistance that may not be discernible through direct visual observation. While incorporating clinical specimens would undoubtedly enhance the value of the study, the heterogeneous nature of cancer cells poses challenges in clearly identifying chemotherapy-resistant cells within a tumor for model training. Despite this limitation, our study serves as a proof-of-concept demonstrating CNN's ability to discern cells with distinct features beyond what is perceptible to the naked eye. We believe that our findings are valuable in various applications; for example, CNN could serve as a tool for monitoring cell properties, thus facilitating quality control during cell culture. We acknowledge the reviewer's concerns regarding the use of a kidney cell line as the control. Therefore, in this revision, we have replaced the images of the kidney cell line with those of normal oral keratinocytes as the control for model training (Page 3, Lines 73, in red).

**Reviewer #2:**

This is a study about the use of deep learning in classification of the morphological shape of oral cancer cells. I have some suggestions to improve this manuscript.

Comment #2-1:

In introduction, add the review of oral cancer cell morphology.

Response #2-1:
We thank the reviewer’s suggestion and have included the review of oral cancer cell morphology (Page 2, Lines 48-53, in red).

Comment #2-2:

In introduction, add the importance of using deep learning in classification of oral cancer cell morphology.

Response #2-2:

We thank the reviewer’s suggestion to elaborate the importance of using deep learning in classification of cancer cell morphology, which lies in its ability to address challenges such as variability in cell morphology and error-prone manual feature extraction. Convolutional Neural Networks (CNN) excel at automatically identifying patterns and structures, making them ideal models for classifying cancer cell morphology, particularly those with subtle changes in cell shape. Moreover, deep learning-based automatic image classification also allows the determination of cell states without cell processing, thus extending its applicability to live cells. We incorporated these aspects, highlighting the importance of deep learning in the classification of cancer cell morphology in the Introduction (Page 3, Lines 40-58, in red).

Comment #2-3:

In Discussion, discuss more about the previous studies in using machine learning/deep learning to classify cancer cell morphology.

Response #2-3:
We thank the reviewer’s suggestion and have elaborated on previous studies that used deep learning for the classification of different types of cell morphology in the Discussion. (Page 14, Lines 424-434, in red).

Comment #2-4:

In Discussion, compare the results of this study to related previous studies in classification of cancer cell morphology using machine learning/ deep learning.

Response #2-4: We thank the reviewer’s suggestion and have incorporated comparisons with other studies, including those that prioritized the speed of the process by reducing the dimensions of the image, integrated masking processes to develop lightweight models, and used the VGG16 model to improve precision. (Page 13, Lines 424-434, in red).

**Reviewer #3:**

Comment #3-1:

The article utilizes convolutional neural networks (CNNs), particularly the EfficientNet-B3 model, to identify and categorize morphological changes in cancer cells, especially those induced by chemotherapy. The study successfully developed and validated a CNN model capable of accurately classifying normal cells, cancer cells, and chemotherapy-resistant cancer cells. Additionally, it can estimate the proportion of chemotherapy-resistant cells within a cell population. This research demonstrates the method's efficacy and precision in recognizing and categorizing cancer cells, particularly those resistant to chemotherapy, offering a novel approach for identifying and classifying subtle cellular morphological changes that are difficult to observe with traditional microscopy techniques. However, while the model exhibits high accuracy, its applicability and validation with actual clinical samples are insufficient. The study lacks an in-depth discussion on the model's applicability and effectiveness across different types of cancer cells. These limitations have also been demonstrated in the discussion section.

Response #3-1:

We thank the reviewer for highlighting the limitation of lacking clinical significance and recommending a discussion on the applicability of the model. We realize that only the model that has been trained with clinical tumor specimens, but not cancer cell lines, can be extended to clinical application. However, training a model to predict patient responses to chemotherapy is challenging because it is essentially impossible to label chemoresistant cancer cells within tumors for model training. Nonetheless, a potential applicability of the model is related to the screening stage of drug development because cancer cell lines are commonly used as a reliable and consistent tool for assessing the efficacy and toxicity of the drugs. Additionally, we also mentioned the value of automated in vitro cell image analysis for real-time assessment of cell state and functionality. We have included these notions in the Discussion (Page 14, Lines 447-461, in red).

Comment #3-2:
Most of the literature cited in the introduction and discussion sections is from studies conducted over three years ago. It is advisable to include more recent references or update the citations.

Response #3-2:
We thank the reviewer’s suggestion and have incorporated more recent references.

Comment #3-3:
Some of the cited literature comprises unreviewed preprints with a low level of evidence. It is recommended to replace these with references from more credible sources.

Response #3-3:
We thank the reviewer’s suggestion and have updated the references.

Comment #3-4:
For the algorithm and modeling section, more explicit details about the hyperparameter tuning process would be beneficial. It's not clear how hyperparameters were optimized and what specific values were used.

Response #3-4:
We thank the reviewer’s suggestion and have summarized the hyperparameter tuning process as well as the hyperparameter setting in a new section “Hyperparameters Setting”. Two tables are also included to increase its clarity. (Page 7, lines 247-280, Table 1 and Table 2, in red).

Comment #3-5:
The caption for Fig 1 requires a period at the end of the sentence.

“Fig 1. Three classes of cells (with labeling), named as HOMO data. Each class has equally 300 images. Notice that the morphological features of three classes of cells have only subtle differences that the expert might be able to distinguish.”

Response #3-5:
We thank the reviewer’s suggestion and have corrected the error in according to the instruction. (Page 4, Fig 1.).

**Reviewer 4**

The research investigates the use of CNN to distinguish between different cell types and hopes to identify chemo-resistant cancer cells based on their morphological features. The manuscript has potential but needs further work to be published. Please revise, clarify, or address the following:

Comment #4-1:
The current title may be construed as misleading since it implies that the image analysis is completed on oral cancer tissue and inflates the clinical significance of the paper. Usage of cell lines for image analysis is also not mentioned at all in the abstract.

Response #4-1:
We thank the reviewer for pointing out that the current title misleads by implying the use of oral cancer tissue for the experiments. We have revised the title to “Deep learning-based automatic image classification of oral cancer cells acquiring chemoresistance in vitro”. We also revised the Abstract to explicitly mention the use of oral cancer cell lines in this study (Page 1, in red).

Comment #4-2:
HEK293T cells (“CONTROL”) – why choose kidney cells for control instead of oral keratinocytes?

Response #4-2:
We thank the reviewer for inquiring. We have replaced the images of the kidney cell line with those of normal oral keratinocytes as the control for model training (Page 3, Lines 73, in red).

Comment #4-3:
To my knowledge, there is no gold-standard in assessing for chemoresistence. Please explain how you overcame this challenge to apply your ground truth labels.

Response #4-3:
We thank the reviewer’s suggestion to clarity the assessment of chemoresistance. In this study, we used low doses of thapsigargin, a known stressor for endoplasmic reticulum (ER), at levels that allowed cancer cells to adapt to stress and gain resistance to chemotherapy. ER stress, commonly driven by oncogenic transformation and hostile tumor microenvironment, is often accumulated in cancer cells. Previous studies have suggested that adaptation to ER stress promotes cell state transition and acquisition of chemoresistance. Indeed, our prior study that used the colorimetric NAD(P)H-based assay to measure the activity of dehydrogenase, a mitochondrial enzyme active only in live cells, has found that the IC50 of cisplatin chemotherapy for OECM1 oral cancer cells increased from 25 µM to 50 µM after cells adapted to low dose thapsigargin. Therefore, while there is no gold-standard for defining chemoresistance, a twofold increase in the IC50 dosage of cisplatin chemotherapy would appropriately indicate the development of chemoresistance. We have included this description in the “Cell culture and image acquisition” section of the Materials and methods. (Page 3, Lines 80-86, in red).

Comment #4-4:
Would be helpful if a table with all the CNN training parameters were included (learning rate, epoch, batch size, ect)

Response #4-4:

We thank the reviewer’s suggestion and have summarized the hyperparameter tuning process as well as the hyperparameter setting in a new section “Hyperparameters Setting”. Two tables are also included to increase its clarity. (Page 7, lines 247-280, Table 1 and Table 2, in red).

Comment #4-5:
The authors reported that the test set showed a perfect score of 100% accuracy. This is unusual unless the data is incredibly homogenous which cell lines may be. Can you add prediction results when the model is applied to different oral cancer cell lines that were not originally trained on? This would be a much better surrogate for the heterogeneity seen in actual patient tissue samples and prove the model’s generalizability - even if accuracy is lower.

Response #4-5:
We acknowledge the reviewer’s concern regarding our result showing 100% accuracy. In this revision, we have replaced the images of the kidney cell line with those of normal oral keratinocytes as the control for model training. The accuracy of the test set of the newly trained models for mixed cell populations (i.e., HETERO data) ranges from 99% to 100% (Table 4). While the test set was not used in the training process, it reached an excellent result because we additionally applied the ensemble learning (EL) method to aggregate all the training model results (from the five-fold cross validation (CV)) before giving a final prediction for each tested image. On the other hand, if we evaluate each trained model (from five-fold CV) on the test set (that is, no EL applied), their average performances are similar to those for validation set, with accuracy ranging from 99.00% to 99.78% (as shown in the table below). As we would like to present the strength of EL, these results are not shown in manuscript but are provided here for reference only). (Page 10, lines 326-331)

| Table. The 5-fold performances (Mean(SE)) of CNN models on test set of HOMO data | | | | | |
| --- | --- | --- | --- | --- | --- |
| **Models**  **Metrics (%)** | **M1.** | **M2.** | **M3.** | **M4.** | **M5.** |
| **Accuracy** | **99.78 (0.30)** | 99.56 (0.72) | 99.11 (0.63) | 99.56 (0.46) | 99.00 (1.07) |
| **Macro Sensitivity** | **99.78 (0.30)** | 99.56 (0.72) | 99.11 (0.63) | 99.56 (0.46) | 99.00 (1.07) |
| **Macro Precision** | **99.78 (0.30)** | 99.57 (0.69) | 99.13 (0.62) | 99.57 (0.45) | 99.05 (0.98) |
| **Macro  F1 score** | **99.78 (0.30)** | 99.56 (0.72) | 99.11 (0.63) | 99.56 (0.46) | 99.00 (1.07) |
| **Macro AUC** | **100.00 (0.00)** | **100.00 (0.00)** | **100.00 (0.00)** | **100.00 (0.00)** | **100.00 (0.00)** |
|  | | | | | |

Comment #4-6:
Conclusion section: line 409 “which is a key feature of cancer cells in response to tumor microenvironment by dynamic cell state transition.” This phrase is confusing. The biologic processes behind tumor heterogeneity are complex and has to do with more than the tumor microenvironment.

Response #4-6:
We thank the reviewer’s comment on the phrase in the conclusion section. We understand that the complexity of tumor heterogeneity arises from multiple factors beyond the tumor microenvironment. Therefore, we revised the sentence to “…, which is a complex interplay of factors including the genetic diversity, tumor microenvironment, and cell adaptation.” (Page 14, Lines 463-465, in red). We hope that this revision more accurately encapsulates the breadth of tumor heterogeneity.

Comment #4-7:
Line 410- What does “dynamic cell state transition” mean?

Response #4-7:
We thank the reviewer for inquiring about the phrase “dynamic cell state transition”. We noticed the unclarity of the entire sentence and have amended it as outlined in Response #4-6 (Page 14, Lines 463-465, in red).

Comment #4-8:
Grad-Cam is used to visualize the predictions but what morphology is expected in chemoresistent cells? Is this matching the Grad-Cam areas?

Response #4-8:
We thank the reviewer’s inquiry regarding Grad-CAM. We used Grad-CAM to visualize model predictions of chemoresistant cell morphology in order to enhance the interpretability of our results. While Grad-CAM primarily highlights regions important for classification, such as cell contours shown in Figure 7, it is important to note that our study focuses on classification accuracy rather than directly predicting the morphology of chemoresistant cells. The heat maps from Grad-CAM effectively demonstrate how our models leverage significant features for classification tasks, which may indirectly correlate with underlying morphological characteristics critical for identifying chemoresistant cells (Page 11, Lines 365-372, in red).
